# Differences Are Important: Breast Cancer Therapy in Different Ethnic Groups

**DOI:** 10.1200/JGO.2017.009936

**Published:** 2017-04-11

**Authors:** Ricardo L.B. Costa, William J. Gradishar

**Affiliations:** **All authors:** Feinberg School of Medicine and Robert H. Lurie Comprehensive Cancer Center of Northwestern University, Chicago, IL

Breast cancer is the most frequently diagnosed cancer and the second leading cause of cancer death among Asian women.^1-3^ Hormonal receptor (HR) –positive tumors are the most common type of breast cancer, and treatment of metastatic disease remains palliative. Endocrine therapy is the cornerstone of treatment of patients with HR-positive metastatic breast cancer (MBC). In postmenopausal patients, aromatase inhibitors have become the treatment of choice in first-line therapy with a median progression-free survival (PFS) of approximately 10 months.^4-7^ Upon disease progression, second-line treatment options include other classes of aromatase inhibitors (steroidal or nonsteroidal), the estrogen receptor antagonist fulvestrant, and tamoxifen, which have modest efficacy (median PFS, 3 to 6 months).^4,8-13^ More recently, further understanding of mechanisms of anti-estrogen therapy resistance (eg, cell cycle kinase aberrations) fostered improvement in MBC therapy. Antiestrogen therapies function partly through suppression of cyclin-dependent kinase 4 (CDK4) and cyclin-dependent kinase 6 (CDK6) activity, and reactivation of these kinases has been implicated in endocrine resistance.^14^ Indeed, in the first-line setting, palbociclib (a small-molecule CDK4/6 inhibitor) has shown efficacy in patients with HR-positive, human epidermal growth factor receptor 2 (HER2) –negative, recurrent or de novo MBC in combination with letrozole. The Palbociclib Ongoing Trials in the Management of Breast Cancer (PALOMA) -2 trial is a double-blind phase III trial in which the median PFS was 24.8 months in the palbociclib plus letrozole group compared with 14.5 months in the placebo plus letrozole group (hazard ratio [HR], 0.58; 95% CI, 0.46 to 0.72; *P* < .001).^15^ In addition, in the Mammary Oncology Assessment of LEE011’s (Ribociclib) Efficacy and Safety (MONALEESA-2) trial, ribociclib (another CDK4/6 inhibitor) also improved the median PFS of patients with HR-positive, HER2-negative, recurrent or de novo MBC who had not received treatment of metastatic disease (HR, 0.56; 95% CI, 0.43 to 0.72; *P* < .001).^16^ In the PALOMA-3 trial, the combination of palbociclib with fulvestrant significantly improved the median PFS in patients with HR-positive, HER2-negative MBC to 9.5 months, compared with 4.6 months among patients treated with fulvestrant and placebo (HR, 0.46; 95% CI, 0.36 to 0.59; *P* < .001).^17,18^

In the article that accompanies this editorial, Iwata et al^19^ report the results of 105 Asian patients enrolled onto the PALOMA-3 trial. This is indeed a relevant preplanned subgroup analysis, because ethnic pharmacogenomic differences pertaining to pharmacokinetics, pharmacodynamics, efficacy, and tolerance are not well understood for CDK inhibitors among Asian patients. Remarkably Asians have been under-represented in other large randomized studies assessing efficacy of CDK inhibitors (ie, MONALEESA-2 trial: 68 of 668 patients were Asian; PALOMA-2 trial: 95 of 666 patients were Asian).^15,16^ The premise of interethnic variability is further corroborated by reports of differential drug metabolism of agents other than palbociclib, such as tamoxifen, through the CYP complex. For instance, as many as 30% of whites are poor metabolizers of tamoxifen given the predominance of the CYP2D6*4 allele (rare among Asians); conversely, 38% to 70% of Asians are intermediate metabolizers of tamoxifen given CYP2D6*10 allele presence (rare among non-Asians).^20,21^ Similarly, polymorphisms in the promoter enhancer region of the *TYMS* gene, which encodes thymidylate synthase, may account for lower capecitabine-induced toxicity rates among Asians compared with whites.^22,23^

Furthermore, outside the realm the pharmacogenomics studies, subgroup analyses of larger breast cancer trials support distinct risk-benefit ratios of selected targeted therapies among Asians with breast cancer. In the Breast Cancer Trials of Oral Everolimus-2 (BOLERO-2), certain toxicities were more common among Asians compared with non-Asians with HR-positive, HER2-negative MBC treated with the mammalian target of rapamycin inhibitor everolimus combined with exemestane, including stomatitis (80% *v* 54%, respectively), rash (50% *v* 37%, respectively), dysgeusia (31% *v* 20%, respectively), and pneumonitis (23% *v* 15%, respectively).^24^ In addition, Asian patients with HER2-positive MBC treated with HER2-targeted antibodies (trastuzumab and pertuzumab) combined with docetaxel needed frequent chemotherapy dose reductions (47% for Asian *v* 13% for non-Asian patients).^25^ A remarkable differential toxicity profile was also observed between Asians and non-Asians (edema, 26% *v* 5%; myalgia, 42% *v* 15%; febrile neutropenia, 19% *v* 7%; upper respiratory tract infection, 26% *v* 10%; decreased appetite, 47% *v* 19%; and rash, 44% *v* 22%, respectively). These examples indicate that there is need to evaluate not only the efficacy, but also the safety of new agents in different ethnic groups, particularly Asian patients, who form a small proportion of early-phase and drug registry clinical trials.

The PALOMA-3 trial showed significant baseline differences between Asians and non-Asians.^19^ For instance, Asians weighed significantly less (mean weight, 57 *v* 75 kg in non-Asians; *P* < .0013) and were shorter (mean height, 156 *v* 163 cm in non-Asians; *P* < .0013). This is remarkable because lower weight (ie, body mass index) has shown positive correlation with an improved clinical benefit rate from fulvestrant for the treatment of HR-positive MBC.^26^

The median PFS in Asians was not reached in the palbociclib arm (95% CI, 9.2 to not reached) but was 5.8 months (95% CI, 3.5 to 9.5 months) in the placebo arm (HR, 0.485; 95% CI, 0.27 to 0.87; *P* = .0065).^19^ Asian patients treated with fulvestrant and placebo had similar PFS to patients in historical Asian and non-Asian controls.^8,27^ In addition, the magnitude of benefit among Asians and non-Asians was similar for the primary end point of PFS in both groups (HR, 0.451; 95% CI, 0.34 to 0.59; *P* < .001 for non-Asians). These efficacy results are in harmony with preliminary analysis of the MONALEESA-2 trial, which enrolled 68 Asians; a preliminary subgroup analysis showed that PFS was significantly prolonged with ribociclib combined with letrozole for patients treated in Asia (HR, 0.298; 95% CI, 0.134 to 0.662) and outside Asia (HR, 0.602; 95% CI, 0.457 to 0.792).^28^

Neutropenia is the most common treatment-related toxicity associated with palbociclib.^29^ In both the PALOMA-2 and PALOMA-3 trials, the most common treatment-related grade 3 or 4 toxicity was neutropenia (66.5% and 65%, respectively). Of note, infection is a rare complication of CDK inhibitor–induced neutropenia, and no deaths were reported as a result of infection in either trial, indicating the favorable safety profile of palbociclib. All patients in the PALOMA-3 trial had trough pharmacokinetic samples for determination of palbociclib plasma concentrations on the first two cycles of treatment, and exposure to treatment was similar between Asians and non-Asians. Palbociclib was well-tolerated among Asians; none of the Asian patients discontinued treatment as a result of toxicity, and measures of patient-reported outcomes showed no significant deterioration in global quality of life. However, Asian patients had higher rates of grade 3 and 4 neutropenia compared with non-Asians (92% *v* 58%, respectively). In a phase I study of palbociclib plus letrozole in Japanese patients, 83% had grade 3 or 4 neutropenia.^30^ Similar results were also seen with ribociclib in the MONALEESA-2 trial, in which grade 3 or 4 neutropenia was documented in 71% of the 35 Asians patients treated with ribociclib and letrozole.^28^ Interestingly, Asians in the PALOMA-3 trial had an absolute neutrophil count 19% lower than non-Asians at baseline, but additional studies are needed understand whether the increased neutropenia rates in Asians are a function of lower pretreatment WBC counts. Among non-Asians, neutropenia has not shown correlation with prior chemotherapy, tumor grade, body weight, or age.^29,31^

Taking into account all caveats inherent to analyses of subpopulations of large clinical trials (eg, invariably small sample size, multiplicity of testing), the data presented by Iwata et al^19^ support the clinically meaningful efficacy of palbociclib for the end point of PFS in Asians. However, this report and others indicate that Asians have a higher risk of adverse events (eg, grade 3 and 4 neutropenia) despite preserved patient-reported outcomes and quality of life; the reasons for this have yet to be elucidated. In light of growing evidence of interethnic pharmacogenomic and safety discrepancies between Asians and non-Asians observed in recently published clinical trials and observational studies, there is a clear need for enhanced enrollment of Asians and other ethnic groups into clinical trials of new agents for the treatment of MBC.
